# AtSWEET4, a hexose facilitator, mediates sugar transport to axial sinks and affects plant development

**DOI:** 10.1038/srep24563

**Published:** 2016-04-22

**Authors:** Xiaozhu Liu, Yan Zhang, Chao Yang, Zhihong Tian, Jianxiong Li

**Affiliations:** 1 Key Laboratory of South China Agricultural Plant Molecular Analysis and Genetic Improvement, and Guangdong Provincial Key Laboratory of Applied Botany, South China Botanical Garden, Chinese Academy of Sciences, Guangzhou 510650, China; 2 College of Life Science, Yangtze University, Jingzhou 434025, China

## Abstract

Plants transport photoassimilates from source organs to sink tissues through the phloem translocation pathway. In the transport phloem, sugars that escape from the sieve tubes are released into the apoplasmic space between the sieve element/companion cell complex (SE/CC) and phloem parenchyma cells (PPCs) during the process of long-distance transport. The competition for sugar acquisition between SE/CC and adjoining PPCs is mediated by plasma membrane translocators. YFP-tagged AtSWEET4 protein is localized in the plasma membrane, and Promoter_*AtSWEET4*_*-GUS* analysis showed that *AtSWEET4* is expressed in the stele of roots and veins of leaves and flowers. Overexpression of *AtSWEET4* in *Arabidopsis* increases plant size and accumulates more glucose and fructose. By contrast, knock-down of *AtSWEET4* by RNA-interference leads to small plant size, reduction in glucose and fructose contents, chlorosis in the leaf vein network, and reduction in chlorophyll content in leaves. Yeast assays demonstrated that AtSWEET4 is able to complement both fructose and glucose transport deficiency. Transgenic plants of *AtSWEET4* overexpression exhibit higher freezing tolerance and support more growth of bacterium *Pseudomonas syringae* pv. *phaseolicola* NPS3121. We conclude that AtSWEET4 plays an important role in mediating sugar transport in axial tissues during plant growth and development.

Photosynthesis of plant green leaves fixes carbon dioxide and synthesizes carbohydrates, which can be transiently stored as starch in mature leaves during the day, but are broken down to sugars and then exported to growing organs as well as for energy metabolism in leaves during the night^1^,^2^. Carbohydrate partitioning within the whole plant is accomplished by phloem transport including the long-distance transport which connects source and sink organs and the short-distance transport which occurs either through plasmodesmata or via highly specific sugar transporters^3^,^4^. A large number of genes encoding different sugar transporters have been identified, for example, more than 53 genes encoding potential monosaccharide transporters and about 20 genes for putative disaccharide carriers were identified in *Arabidopsis*^5^,^6^,^7^. Different plants use different phloem loading mechanisms to translocate sugars into the phloem transport stream. In the well-known phloem loading mechanism, the companion cells are isolated from surrounding cells and require transporters to import sugars for translocation^8^. For example, AtSUC2 is a phloem-specific plasma membrane sucrose transporter and required for phloem loading of sucrose in leaves^9^,^10^. Loss of AtSUC2 results in stunted growth, retarded development, and sterility in *Arabidopsis*^11^.

In addition to long-distance transport of sugars, there is some consensus that the release and retrieval mechanisms along the phloem transport pathway result in a sugar exchange between the sieve element/companion cell complex (SE/CC) and the adjacent phloem parenchyma cells (PPCs)^12^,^13^. Allocation of sugars between SE/CC and PPCs depends on the respective membrane-bound transporters. It has been reported that the plasma membrane is equipped with a multitude of sugar transporters^10^,^14^, and much more attention has been put on studying their roles in long-distance phloem transport than in axial transport of sugars. The axial release/retrieval of the transporting sugars must be tightly controlled because it influences the mass flow rates of solutes through the phloem and finally affects plant growth.

SWEETs are sugar transporters, which are present in plants as well as in animals and humans^15^. *Arabidopsis* contains 17 *SWEET* genes. AtSWEET11 and 12 localize to the plasma membrane and mediate sucrose export from phloem parenchyma cells into the apoplasmic space to supply sucrose for the H^+^-coupled sugar transporter SUT1 in the SE/CC^16^. Single knockout mutants of *AtSWEET11* and *12* did not display any obvious altered morphological phenotype, whereas double mutant plants showed an expected phenotype of blocked phloem loading, i.e., smaller plants, elevated levels of leaf starch, a reduced export of fixed ^14^C from leaves and reduced growth of roots^16^.

SWEET9 functions as a nectary-specific sucrose transporter^17^. Starch-derived sucrose is synthesized by sucrose phosphate synthase and exported by SWEET9, leading to sucrose accumulation in the apoplasm, where sucrose is hydrolysed by an apoplasmic invertase to produce a mixture of sucrose, glucose and fructose^17^. AtSWEET16 is a vacuole-located sugar transporter, which is able to transport glucose, fructose, and sucrose when expressed in heterologous *Xenopus laevis* oocytes^7^. *Arabidopsis* transgenic plants overexpressing *AtSWEET16* showed altered germination rate, growth phenotype, and stress tolerance^7^. *AtSWEET17* is a vacuolar fructose exporter^18^. It is highly expressed in the cortex of roots and functions as an energy-independent fructose carrier, overexpression of *AtSWEET17* specifically reduces the fructose content in leaves^19^. Recently, maize ZmSWEET4c was demonstrated to mediate transepithelial hexose (e.g. fructose and glucose), but not the sucrose, transport across the basal endosperm transfer layer into the seed, and rice OsSWEET4 functions as a glucose and fructose transporter^20^.

SWEET-mediated sugar transport is not only essential for carbohydrate distribution but also for pathogen resistance. Grapevine (*Vitis vinifera*) SWEET4 (VvSWEET4) is a plasma membrane protein and acts as a glucose transporter^21^. The expression levels of *AtSWEET4* and *VvSWEET4* were induced upon *Botritis cinerea* infection^15^,^21^, and the *Arabidopsis* knockout mutants in the orthologous *AtSWEET4*, *atsweet4-1* and -*2*, showed enhanced resistance to *B. cinerea* infection^21^.

Here, we report the characteristics of *AtSWEET4*. We studied the cellular localization of AtSWEET4 and analyzed its tissue specific expression patterns. We generated *AtSWEET4* overexpression and knock-down transgenic *Arabidopsis* lines and studied the phenotypic changes in transgenic plants with altered *AtSWEET4* expression. Knock-down of *AtSWEET4* expression led to less glucose and fructose contents and chlorosis along the vein network. Our results suggest that AtSWEET4 plays a key role in mediating sugar supply to the axial tissues, and that the tight control of *AtSWEET4* expression is important for plant growth.

## Results

### Altered growth phenotypes in *AtSWEET4* RNA-interference and overexpression lines

**Figure 1:**
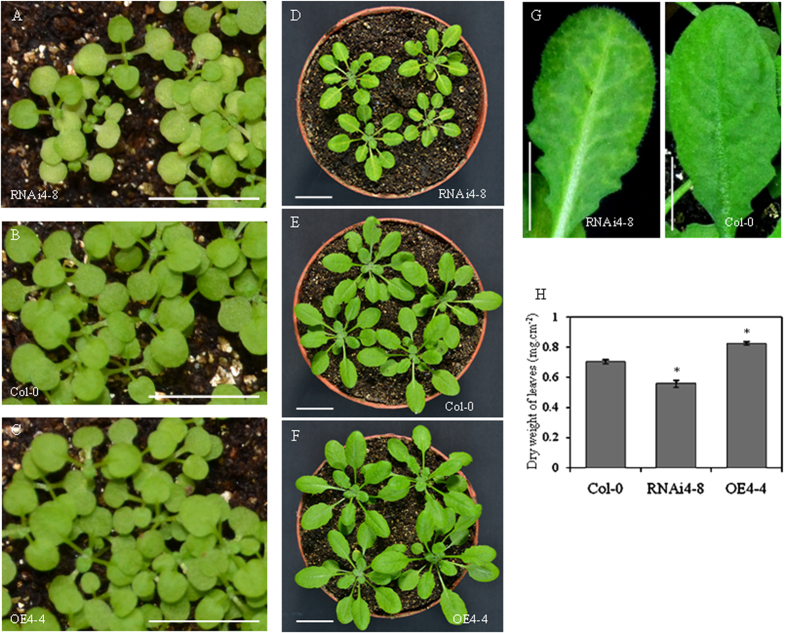
Phenotypes of *AtSWEET4* transgenic plants. Phenotypes of 10-day-old seedlings of *AtSWEET4*-RNAi transgenic line RNAi4-8 (**A**), wild-type Col-0 (**B**), and *AtSWEET4* overexpression line OE4-4 (**C**). Phenotypes of 3-week-old plants of RNAi4-8 (**D**), Col-0 (**E**), and OE 4-4 (**F**) grown in soil. (**G**) Mature leaves of RNAi4-8 showed chlorosis in the vein network. Scale bars: 1 cm. (**H**) Dry weight of leaves from Col-0, RNAi4-8, and OE4-4. Thirty leaves were collected from each type of plants, the area and weight were measured for each leaf, after drying at 85 °C in oven for two days, each leaf was weighed again. Data represent the mean ± SE (n = 3), *p value < 0.05, t-test.

Previous studies indicated that SWEET genes are genetically redundant, thus single knockout mutants fail to show a noticeable phenotype^6^,^16^. To investigate the physiological function of AtSWEET4, we generated *AtSWEET4* knock-down (RNAi lines) and overexpression (OE lines) transgenic plants. In total, 11 out of 17 positive RNAi transgenic plants showed reduced amounts of *AtSWEET4* mRNA and leaf chlorosis, and 12 *AtSWEET4* overexpression lines were obtained. Homologous transgenic plants RNAi4-8 and OE4-4 with single T-DNA insertion, respectively, were chosen for further analyses. We observed that, compared to wild-type Col-0 plants, both RNAi4-8 lines and OE4-4 transgenic plants showed altered growth phenotypes (Fig. 1A–F).

Under normal growth conditions, the seedlings of RNAi4-8 displayed yellow cotyledons, whereas OE4-4 seedlings were as green as the wild-type (Fig. 1A–C). At the later stages of growth, mature leaves of RNAi4-8 showed chlorosis along the vein network and were green in the interveinal regions (Fig. 1G). Generally, the plant size of RNAi4-8 was smaller than that of wild-type Col-0 (Fig. 1D,E), and OE4-4 plants were larger than wild-type Col-0 (Fig. 1E,F). Furthermore, the leaf size of RNAi4-8 was smaller than that of wild-type Col-0, by contrast, OE4-4 plants had larger leaf size than wild-type plants (Supplementary Fig. 1A). The different plant sizes of these three plants were also pinpointed by biomass accumulation. The 4-week-old plants of RNAi4-8, OE4-4, and Col-0 grown in soil under the normal conditions were used for biomass assessment. The dry biomass of Col-0 plants was significantly different to that of RNAi4-8 and OE4-4 plants in that Col-0 plants showed a dry biomass of 0.70 mg per square centimeter, whereas RNAi4-8 and OE4-4 exhibited 0.56 mg and 0.83 mg dry biomasses, respectively (Fig. 1H). In addition, the fresh biomasses of these three types of plants were also calculated and showed difference (Supplementary Fig. 1B).

### Genetic modulation of *AtSWEET4* expression leads to phenotypic alteration

**Figure 2:**
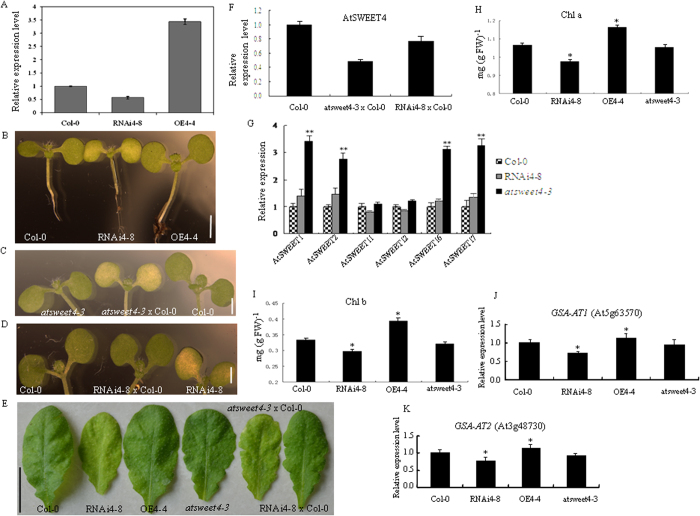
Characterization of *AtSWEET4*. (**A**) Relative expression levels of *AtSWEET4* in wild-type Col-0, RNAi4-8, and OE4-4 plants detected by qRT-PCR. (**B**) Different sizes of wild-type Col-0, RNAi4-8, and OE4-4 seedlings grown in soil under normal conditions for 5 days. (**C**) Phenotype of F_1_ seedlings from the cross of *atsweet4-3* and Col-0. (**D**) Phenotype of F_1_ seedlings from the cross of Col-0 and RNAi4-8. (**E**) Phenotypes of mature leaves detached from Col-0, RNA4-8, OE4-4, *atsweet4-3*, and F_1_’s of *atsweet4-3 *× Col-0 and RNAi4-8 × Col-0. (**F**) qRT-PCR analysis of AtSWEET4 expression in Col-0 and F1’s from crosses of *atsweet4-3* × Col-0 and RNAi4-8 × Col-0. (**G**) Expression levels of six other *AtSWEET* genes in Col-0, RNAi4-8, and *atsweet4-3* plants. Data represent the mean ± SE (n = 3). **p value < 0.01, t-test. (**H**,**I**) Chlorophyll a content (**H**) and Chlorophyll b content (**I**) in Col-0, *AtSWEET4*-transgenic lines, and *atsweet4-3* plants. Fully expanded rosette leaves were collected at the daytime from each type of plants and weighed for 500 mg, strips of leaves were used for chlorophyll measurement, and three replicates were performed. Data represent the mean ± SE (n = 3). *p value < 0.05, t-test. (**J**,**K**) The expression levels of glutamate semialdehyde aminotransferase 1 gene (*GSA-AT1*) (**J**) and *GSA-AT2* (**K**) in Col-0, RNAi4-8, OE4-4, and *atsweet4-3* plants. Data represent the mean ± SE (n = 3). *p value < 0.05, t-test. Scale bars: 1 mm (**B**–**D**) and 1 cm (**E**).

Since RNAi4-8 and OE4-4 transgenic plants displayed phenotypic alterations, we used quantitative RT-PCR (qRT-PCR) to investigate the expression levels of *AtSWEET4* in these two lines (Fig. 2A), and other RNAi and OE lines (Supplementary Fig. 1C). RNA-interference reduced *AtSWEET4* level in RNAi4-8 to half of the wild-type expression level, whereas overexpression resulted in a 2-fold increase in *AtSWEET4* level in OE4-4 (Fig. 2A).

Overexpression and knock-down of *AtSWEET4* expression led to developmental alterations observed as early as in the seedling stage in which RNAi4-8 seedlings showed smaller size and chlorosis in cotyledon, whereas OE4-4 displayed larger cotyledon and longer hypocotyl (Fig. 2B). To study the function of AtSWEET4 in detail, a homologous knockout mutant *atsweet4-3* with T-DNA insertion in the sixth exon was obtained (Supplementary Fig. 2A), the *atsweet4-3* mutant did not show obvious altered phenotype when compared to the wild-type Col-0 (Fig. 2C, Supplementary Fig. 2B,C), which is consistent with the findings of Chong *et al.*^21^. Analysis of RT-PCR showed there is no *AtSWEET4* mRNA in *atsweet4-3* mutant (Supplementary Fig. 2D). The phenotypic difference between *atsweet4-3* mutant and RNAi4-8 transgenic line intrigued us to postulate that the phenotypic inconsistency might be caused by the different expression levels of *AtSWEET4* in *atsweet4-3* mutant and RNAi4-8 line. To this end, we crossed *atsweet4-3* mutant with wild-type Col-0, as expected, the cotyledons of F_1_ seedlings from this cross showed chlorosis which resembled the phenotype of RNAi4-8 seedlings (Fig. 2C). Furthermore, in another cross of RNAi4-8 and Col-0 in which Col-0 was used as pollen donor, the F_1_ seedlings from this cross exhibited green cotyledons, rescuing RNAi4-8 phenotype to the wild-type (Fig. 2D). At later stages of plant growth, the mature leaves of F_1_’s from the cross of *atsweet4-3* and Col-0 showed chlorosis phenotype similar to that of RNAi4-8, and leaves from the cross of RNAi4-8 and Col-0 were as green as the wild-type plants (Fig. 2E). We used qRT-PCR to investigate the expression levels of *AtSWEET4* in these F1’s, and the results indicated that the expression level *AtSWEET4* in F_1_’s from the cross of *atsweet4* and Col-0 was reduced to half of the wild-type expression level in Col-0 (Fig. 2F). Expression of *AtSWEET4* in the F_1_’s of RNAi4-8 × Col-0 cross was about 75% of that of the wild-type plants (Fig. 2F). These results indicate that there is a dosage effect on the expression level of *AtSWEET4*, and the amount of *AtSWEET4* mRNA is critical to plant phenotype. In addition, we also crossed RNAi4-8 line and *atsweet4-3* mutant with OE4-4 plants, respectively, using OE4-4 as pollen donor, all F_1_’s from these two crosses showed green cotyledons, resembling the phenotype of OE4-4 (Supplementary Fig. 2E,F). We examined the expression levels of other *AtSWEET* genes in Col-0, RNAi4-8, and *atsweet4-3* plants, among six genes tested, four of them (*AtSWEET1*, *2*, *16,* and *17*) showed significant expression increase in *atsweet4-3* plants, and all six genes did not show expression difference between Col-0 and RNAi4-8 plants (Fig. 2G).

Leaf chlorosis may result from changes in chlorophyll content. We measured the chlorophyll contents in RNAi4-8, OE4-4, and *atsweet4-3* mutant. Down-regulation of *AtSWEET4* expression in RNAi4-8 reduced chlorophyll a/b contents, by contrast, overexpression of *AtSWEET4* in OE4-4 increased chlorophyll a/b contents, while *atsweet4-3* mutant and wild-type Col-0 did not show difference in chlorophyll a/b contents (Fig. 2H,I). Biosynthesis of chlorophyll involves many steps. Glutamate semialdehyde aminotransferase (GSA-AT) catalyzes the last step in the conversion of glutamate to δ–aminolevulinate which is an important intermediate molecule for chlorophyll biosynthesis. We investigated the expression levels of two *GSA-AT* genes in *AtSWEET4* transgenic, *atsweet4-3* mutant and wild-type plants, qRT-PCR analysis showed that expression levels of *GSA-AT1* and *GSA-AT2* were reduced in RNAi4-8 and increased in OE4-4 when compared to their expression in Col-0, there was no significant difference in the expression of these two genes between *atsweet4-3* and Col-0 (Fig. 2J,K).

Taken together, these results indicate that variations on *AtSWEET4* expression result in different phenotypic alterations. Phenotypes of yellow cotyledon and chlorosis in mature leaves are related to a specific *AtSWEET4* expression level which equals to half level of *AtSWEET4* in the wild-type Col-0. However, null expression of *AtSWEET4* does not cause obvious phenotypic change. Suppression of *AtSWEET4* expression in RNAi4-8 leads to reductions in the expression levels of *GSA-AT* genes and decreases in chlorophyll contents, which consequently result in chlorosis in leaves.

### AtSWEET4 mediates fructose and glucose transport

**Figure 3:**
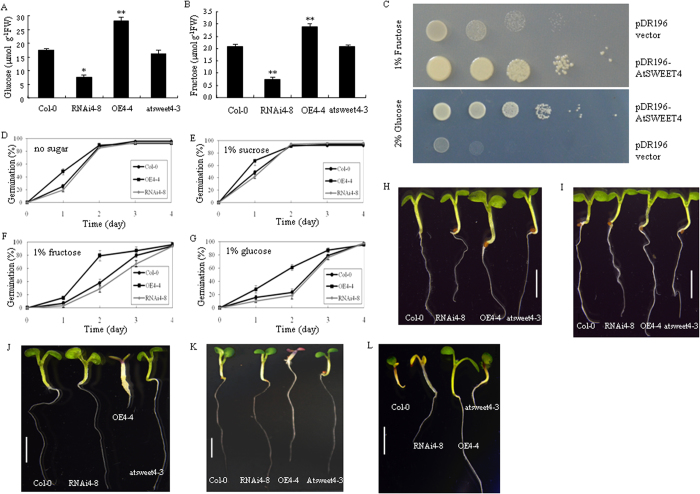
AtSWEET4 mediates glucose and fructose transport. Glucose (**A**) and fructose (**B**) contents in the leaves of wild-type Col-0, transgenic lines, and *atsweet4-3* plants. Fully expanded rosette leaves were collected at the end of night and weighed for 50 mg, extracted with extraction buffers, and then performed for gas chromatograph-mass spectrometry (GC-MS) analysis. Each analysis was performed three times with similar results. The data represent the mean ± SE (n = 3). *p value < 0.05, **p value < 0.01, t-test. (**C**) Complementation of fructose and glucose transport deficiency by AtSWEET4 in yeast EBY.VW4000 strain. (**D**–**G**) Germination rates of Col-0, RNAi4-8, and OE4-4 on 1/2 MS (1/2 MS salts) media supplemented with no sugar (**D**), 1% sucrose (**E**), 1% fructose (**F**), and 1% glucose (**G**). Sixty seedlings were counted for each plant. (**H**–**K**) Phenotypes of seedlings of Col-0, RNAi4-8, OE4-4, and *atsweet4-3* on 1/2 MS media supplemented with 1% fructose (**H**), 1% glucose (**I**), 6% fructose (**J**), and 6% glucose (**K**). (**L**) Responses of seedlings to ABA, seedlings of Col-0, RNAi4-8, OE4-4, and *atsweet4-3* were grown on 1/2 MS media supplemented with 1 μM ABA. Scale bars: 0.5 cm.

SWEETs are able to transport sugars. Phenotypic alterations in RNAi4-8 and OE4-4 may result from different sugar contents. When grown in soil under normal conditions, RNAi4-8 plants accumulated significantly less glucose and fructose in leaves than wild-type plants, whereas OE4-4 accumulated significantly more glucose and fructose than wild-type plants, indicating that AtSWEET4 is able to import both glucose and fructose (Fig. 3A,B). However, *atsweet4-3* mutant and wild-type plants did not show significant difference in glucose and fructose contents (Fig. 3A,B). To further confirm the capability that AtSWEET4 is able to transport glucose and fructose, we performed a yeast complementation assay. The results showed that AtSWEET4 complemented the glucose and fructose uptake deficiency of the EBY.VW4000 yeast strain (Fig. 3C).

Next, we monitored the germination efficiency and seedling growth of Col-0, RNAi4-8, and OE4-4 on half-strength MS (1/2 MS salts) media supplemented with fructose, glucose, and sucrose, respectively.After 1 day (d) of germination on 1/2 MS media without sugar, OE4-4 developed faster than Col-0, whereas RNAi4-8 and Col-0 plants showed similar germination efficiency (Fig. 3D). At the following three days, all three lines showed almost the same germination rate (Fig. 3D). Addition of 1% sucrose to media provoked an increase in germination efficiency for all three lines at 1 dag (day after germination) (Fig. 3E). At 2 dag, seeds from all three lines already completed this first developmental phase and reached 95% germination rate (Fig. 3E). Both glucose and fructose inhibited germination efficiency of all three lines, at 1 dag, fructose showed more severe inhibition than glucose (Fig. 3F,G). However, at 2 dag, fructose had less inhibition efficiency than glucose, in this case OE4-4 was much less sensitive to both glucose and fructose than RNAi4-8 and Col-0 (Fig. 3F,G). At 3 dag, fructose and glucose did not show much difference in inhibition efficiency although the inhibition still existed, and at 4 dag, seeds of all three lines reached 98% germination rate (Fig. 3F,G).

The growth of seedlings from OE4-4, RNAi4-8, *atsweet4-3,* and Col-0 on 1/2 MS supplemented with different concentrations of fructose or glucose was observed. Compared to the seedlings grown in soil, addition of 1% fructose did not have much effect on seedling growth except that it rescued the yellow cotyledons of RNAi4-8 seedlings (Figs 2B and 3H). By contrast, addition of 1% glucose not only rescued the yellow cotyledons of RNAi4-8 but also suppressed the growth of OE4-4 (Fig. 3I). Increase in concentration of fructose and glucose aggravated the inhibitory effect on the growth of OE4-4 seedlings. Addition of 6% fructose severely inhibited the growth of OE4-4 seedlings, as shown in Fig. 3J, OE4-4 seedlings showed small and yellow cotyledons, thick and white hypocotyls, and very short roots. Addition of 6% glucose also suppressed the growth of OE4-4 seedlings, but the inhibitory effect of 6% glucose is different to that of 6% fructose. OE4-4 seedlings grown on the media supplemented with 6% glucose showed purple and small cotyledons and shortened hypocotyls (Fig. 3K). The responses of seedlings to different concentrations of glucose and fructose were not due to high osmotic effects, because all seedlings grew similarly on 1% and 6% sorbitol agar media with 1/2 MS salts, respectively (Supplementary Fig. 3A,B).

ABA was reported to play roles in sugar-mediated signal transduction^22^. We analyzed the responses of these four types of seedlings to ABA treatment. Col-0 and *atsweet4-3* seedlings displayed developmental arrest at a level of 1 μM ABA, and RNAi4-8 seedlings exhibited severely stressed phenotype, whereas OE4-4 seedlings did not show too much growth inhibition in the presence of 1 μM ABA (Fig. 3L). A high level of 2 μM ABA eventually suppressed the growth of OE4-4 seedlings (Supplementary Fig. 3C). These results suggest that AtSWEET4-facilitated fructose and glucose import may enhance tolerance to exogenous ABA stress.

Starch is synthesized and stored in the mature leaves during the day and broken down to sugars during the night. AtSWEET11 and 12 have been shown to export sucrose and are responsible for phloem loading^16^. To explore the possible role of AtSWEET4 in these processes, we examined the starch accumulation and degradation in *AtSWEET4* transgenic plants, starch-staining showed that there is no difference in starch degradation among RNAi4-8, OE4-4, and Col-0 at the end of night (Supplementary Fig. 3D). RNAi4-8 accumulated a little less carbohydrates than OE4-4 and Col-0 at the end of light, which may be caused by the lower chlorophyll content (Fig. 2H,I).

### *AtSWEET4* mediates freezing and drought tolerance and nonhost resistance

**Figure 4:**
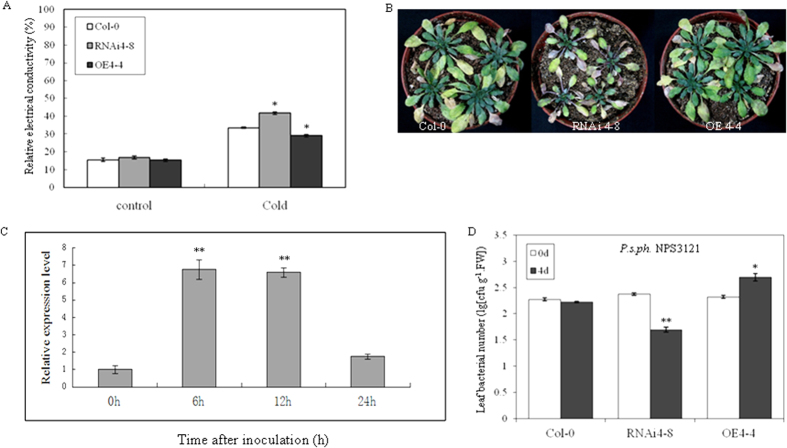
*AtSWEET4* mediates stress responses. (**A**) Overexpression of *AtSWEET4* protects plant cells from being damaged under freezing treatment. Leaves were collected from three types of plants and measured for relative electrolyte leakage; each experiment was carried out with three replicates. Data represent the mean ± SE (n = 3). *p value < 0.05, t-test. (**B**) Reduction in *AtSWEET4* expression leads to sensitivity to drought treatment, the 4-week-old plants of Col-0, RNAi4-8, and OE4-4 grown in soil were withheld water for 23 days when pictures were taken. (**C**) Expression level of *AtSWEET4* was induced by infection of nonhost bacterium *Pseudomonas syringae* pv*. phaseolicola* NPS3121. Data represent the mean ± SE (n = 3). **p value < 0.01, t-test. (**D**) Growth of nonhost bacterium *Psph* NPS3121 strain in the leaves of 5-week-old plants after infiltration with the inoculum. The experiments were carried out in duplicate, each with four plants, data represent the mean ± SE. *p value < 0.05, **p value < 0.01, t-test.

Accumulation of high levels of soluble sugars in the cold is part of a complex metabolic reprogramming in plants, which is expected to help tolerate low temperatures. Thus, we were interested in knowing whether changes in *AtSWEET4* expression had effects on freezing tolerance. The 4-week-old plants of Col-0, RNAi4-8, and OE4-4 were treated under freezing temperature (−6 °C) for 3 hours, leaves from three types of plants were detached to quantify the release of electrolytes, which is an indicator for the amount of destroyed cells^23^. Under normal conditions, the relative electrical conductivities of leaves from these three types of plants were not different, but under freezing conditions RNAi4-8 and OE4-4 showed significantly higher and lower relative electrical conductivities than the wild-type plants, respectively (Fig. 4A), indicating that higher concentration of glucose and/or fructose can protect plant cells from being damaged under freezing condition.

Increase in accumulation of compatible solutes such as glucose and fructose can improve drought tolerance in plants. The 4-week-old plants of RNAi4-8, OE4-4, and Col-0 grown in soil were withheld water for 23 days, after drought treatment, RNAi4-8 plants showed drought-stressed phenotypes of chlorosis and purple leaves, OE4-4 and Col-0 plants were also stressed but to a less extent (Fig. 4B), suggesting that decrease in *AtSWEET4* expression level reduces drought tolerance in plants.

To investigate the potential role of *AtSWEET4* in bacterial disease resistance, we monitored the expression levels of *AtSWEET4* in Col-0 leaves after inoculation with nonhost bacterium *Pseudomonas syringae* pv. *phaseolicola* (*Psph*) NPS3121. mRNA levels of *AtSWEET4* were highly induced by *Psph* NPS3121 infection at 6 and 12 hours after inoculation (Fig. 4C). Furthermore, *Psph* NPS3121 bacterial growth was measured in Col-0, RNAi4-8, and OE4-4 plants 4 days following inoculation. Bacterial population was significantly increased in OE4-4 when compared to Col-0, by contrast, RNAi4-8 transgenic plants supported much less nonhost bacterial growth (Fig. 4D). These results suggest that increase in *AtSWEET4* expression leads to being more susceptible to the nonhost bacterial infection.

### Tissue specificity of *AtSWEET4* expression during plant development

Previous reports indicated that the expression levels of most SWEET genes appear to be very low^7^,^18^,^19^. Quantitative RT-PCR analysis revealed that *AtSWEET4* gene was expressed in many tissues except the siliques during plant development (Supplementary Fig. 4A).

**Figure 5:**
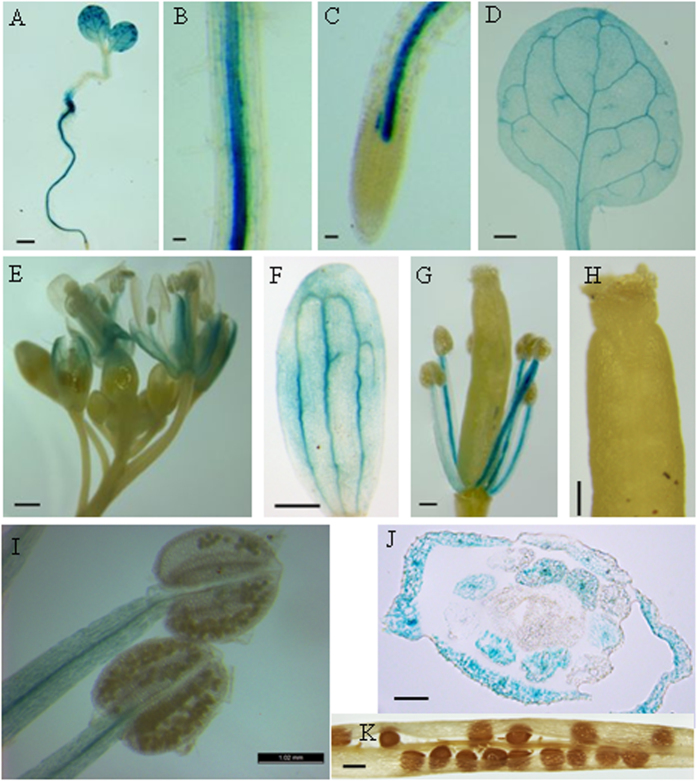
Histological expression profiles of *AtSWEET4*. A construct of Pro_*AtSWEET4*_*-GUS* was expressed in *Arabidopsis* plants and GUS activity was monitored during the reporter plant development. (**A**) A five-day-old seedling showing GUS staining. (**B**) GUS activity in the stele of roots. (**C**) GUS activity detected in mature zone of roots but not in root tip. (**D**) GUS staining detected in the leaf vein network. (**E**) GUS staining detected in mature flowers but not in young flower buds. (**F**) GUS staining in petal vein network. (**G**) GUS activity in anther filaments. (**H**) GUS staining is not detected in pistil. (**I**) GUS staining is detected in anther filaments but not in anthers. (**J**) GUS activity in the cross section of a mature flower. (**K**) GUS staining is not detected in seeds and silique. Scale bars: 1 mm.

To further investigate the expression profile of *AtSWEET4* gene, we generated transgenic reporter plants expressing *GUS* gene under the control of the *AtSWEET4* promoter. Analysis of the reporter plants revealed *AtSWEET4* promoter activity in different tissues during plant development. In 7-day-old transgenic seedlings, GUS activity was mainly detected in cotyledons and roots (Fig. 5A). However, a closer view of GUS activity in roots showed that GUS staining was mainly observed in the stele of roots but not in the cortex and root tips (Fig. 5B,C). In young transgenic plants, GUS activity was also detected in young rosette leaves, but mainly restricted to the vein network, including minor and major veins (Fig. 5D).

At flowering stage, GUS activity was mainly detected in mature flowers and very low GUS activity was detected in young flower buds (Fig. 5E). In mature flowers, GUS activity was largely observed along the vein network of petals (Fig. 5F) and in the anther filaments (Fig. 5G). By contrast, the anthers and pistil did not show GUS activity (Fig. 5H,I). Cross-sections of mature flowers histochemically stained for GUS activity further demonstrated that AtSWEET4 accumulated in the petals and anther filaments but not in the pistil of flowers (Fig. 5J). Furthermore, GUS activity was not detected in seeds and siliques (Fig. 5K).

### AtSWEET4 is a membrane protein

**Figure 6:**
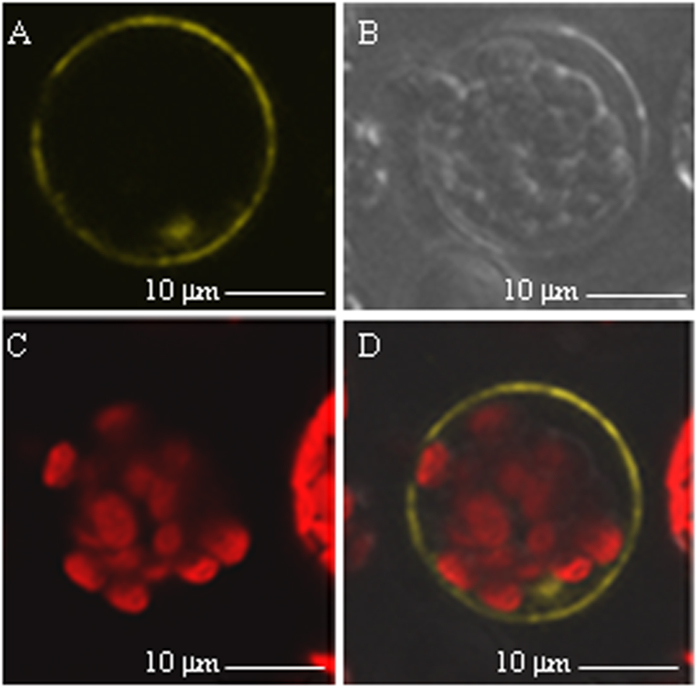
Subcellular localization of AtSWEET4. The plasmid containing 35S_pro_*-AtSWEET4-YFP* fusion was introduced into *Arabidopsis* protoplasts, AtSWEET4-YFP fusion protein localizes in the plasma membrane. (**A**) YFP fluorescence detected in the plasma membrane. (**B**) Bright field of protoplasts. (**C**) Red autofluorescence of the chloroplasts. (**D**) Image merged from (**A**–**C**).

Previous reports showed that different AtSWEETs have different cellular localizations^7^,^15^,^18^. *VvSWEET4*, the closest homolog of *AtSWEET4* in grapevine, was demonstrated to encode a plasma membrane protein^21^. To investigate the cellular localization of AtSWEET4, we made a 35S_Pro_-*AtSWEET4-YFP* construct and transfected the isolated *Arabidopsis* protoplasts. Confocal imaging of AtSWEET4-YFP fusion protein indicated that AtSWEET4 is located in the plasma membrane (Fig. 6A). Bright field showed the intact protoplasts (Fig. 6B), and the red-fluorescence indicated the positions of chloroplasts (Fig. 6C). The merged imagine was shown in Fig. 6D.

## Discussion

Different expression levels of *AtSWEET4* are correlated to different phenotypic alterations. Overexpression of *AtSWEET4* in *Arabidopsis* plants leads to increased plant size observed not only at the mature stage but also at the young seedling stage (Figs 1 and 2). Reduction in *AtSWEET4* expression level leads to phenotypic alterations (Fig. 2), but this reduction has a critical threshold (i.e. half of the wild-type expression level), changes of *AtSWEET4* expression levels above or below this threshold lead to no visible phenotypic alterations (Fig. 2, Supplementary Fig. 2).

We demonstrated that the phenotypic discrepancy between *atsweet4-3* null mutant and RNAi transgenic line RNAi4-8 is caused by the different expression levels of *AtSWEET4*. We obtained 11 independent transgenic lines showing leaf chlorosis. All these lines were examined by qRT-PCR to show reduced amounts of *AtSWEET4* mRNA. These independent transgenic lines exclude the possibility that the phenotypic change of RNAi4-8 is caused by the disruption of other genes rather than interfere with the expression of *AtSWEET4*. Strong evidence comes from the cross of *atsweet4-3* mutant and Col-0, *atsweet4-3* is a null mutant in which *AtSWEET4* gene was knocked out (Supplementary Fig. 2), the F_1_ progeny from this cross exhibited chlorosis phenotype similar to that of RNAi4-8 (Fig. 2). This result demonstrates that variations in *AtSWEET4* expression are responsible for the phenotypic alterations, in addition, it also excludes the possibility that the phenotypic alteration in RNAi4-8 was caused by the secondary effects of RNAi technology. Finally, AtSWEET4 shares 58% of sequence identity with AtSWEET5^15^, interfering with the expression of *AtSWEET4* might also have an impact on the expression of *AtSWEET5*. To exclude this possibility, we examined the expression levels of *AtSWEET5* in the flowers of RNAi4-8, OE4-4, and Col-0 plants because previous reports showed that *AtSWEET5* is not expressed in other tissues except in pollens^16^,^19^,^24^, and no difference in *AtSWEET5* expression was detected (Supplementary Fig. 4B).

It was reported that the *Arabidopsis* genome has 17 SWEET members^15^, there might be some functional redundancy between AtSWEET members, and loss of AtSWEET4 in *atsweet4-3* mutant may be functionally complemented by other AtSWEETs, thus, *atsweet4-3* appears to be a wild-type. In other cases, if AtSWEET4 is still functional but only reduced in amounts, other AtSWEETs may not compensate for the reduced function of AtSWEET4. To this end, we analyzed the expression levels of *AtSWEET1*, *2*, *11*, *12, 16,* and *17* genes in RNAi4-8, *atsweet4-3,* and Col-0 plants, qRT-PCR results showed that all these six AtSWEET genes did not show expression difference in RNAi4-8 and Col-0 plants, interestingly, the expression levels of four AtSWEET genes, *AtSWEET1*, *2, 16,* and *17* were significantly increased in *atsweet4-3* mutant (Fig. 2G). Previous studies showed that AtSWEET1 is a glucose uniporter^15^, AtSWEET16 is responsible for sugar accumulation such as glucose, fructose, and sucrose^7^, and AtSWEET17 is a fructose transporter^18^. A recent study showed AtSWEET2 contributes to the accumulation of glucose *in planta*^25^. These results support our assumption that other AtSWEETs compensate for the lost function of AtSWEET4, thus *atsweet4-3* mutant looks like a wild-type. In addition, *AtSWEET4* expression might have a critical threshold (half of the wild-type expression level) that determines the function of AtSWEET4 and affects plant development.

Carbohydrate distribution is important for plant development. In transport phloem, photoassimilates that escape from the sieve tubes are released into the apoplasmic space between SE/CC and PPCs, allocation of the photoassimilates is mediated by the membrane-bound sugar translocators^13^. Photoassimilates from sources support the maintenance and growth of tissues in both terminal sinks and axial sinks^26^, and expression changes in the sugar translocator genes leads to phenotypic alterations. In tobacco, NtSUT1 was confirmed to be essential for sucrose export from leaves, *NtSUT1* antisense transgenic tobacco plants showed small size and developed chlorosis in leaves^14^. The mature leaves of *NtSUT1* antisense tobacco plants exhibited a progressive development of chlorosis in the interveinal regions and followed a sink-to-source transition pattern starting from the leaf tips and moving to the base as the leaves developed^14^. In addition, functional characterization of SUT1 in *Arabidopsis* (also called AtSUC2) revealed that it is essential for phloem loading but not for long-distance transport^10^. The *AtSWEET4* RNA-interference RNAi4-8 showed chlorosis along the vein network but were green in the interveinal regions, which is typically opposite to the phenotype observed in *NtSUT1* antisense transgenic tobacco plants, implying that AtSWEET4 may mediate sugar import and function in phloem unloading which is essential for axial sugar transport during plant development. Thus, reduced expression of *AtSWEET4* decreases contents of sugars in axial tissues and affects plant growth.

Chlorophyll, including chlorophyll a and b, is the main photosynthetic pigment, its amount directly affects plant photosynthetic efficiency, and increased chlorophyll content leads to increases in biomass production and grain yield^27^. Down-regulation of *AtSWEET4* in RNAi4-8 plants results in decreases in chlorophyll a and b content (Fig. 2H,I), which may reduce the photosynthetic efficiency, thus leading to reduced wet- and dry-biomasses and smaller plant size; on the contrary, OE4-4 plants may have higher photosynthetic efficiency, leading to increased wet- and dry-biomasses and larger plant size. Yeast complementation assays demonstrated that AtSWEET4 is a sugar facilitator for glucose and fructose. Down-regulation of *AtSWEET4* in RNAi4-8 leads to reduced content of glucose and fructose. Glucose is an initial substrate for glycolysis, and the intermediates of glycolysis such as pyruvate and glyceraldehydes-3-phosphate are initial substrates for chlorophyll biosynthesis^28^. Since chlorophyll biosynthesis is tightly controlled in adaptation to environmental factors and developmental program, substrate limitation may down-regulate the expression level of chlorophyll biosynthetic genes such as GSA-AT (Glutamate semialdehyde aminotransferase), thus, leading to reduced chlorophyll content^29^,^30^. Interestingly, tobacco transgenic plants expressing antisense *GSA-AT* showed chlorosis in the areas close to the leaf veins and the stems^29^, which is similar to the phenotype observed in RNAi4-8.

*AtSWEET5* is the closest homolog of *AtSWEET4* in *Arabidopsis*. *AtSWEET5* promoter analysis demonstrated that *AtSWEET5* is active in the vegetative cell during the later stages of pollen development^24^. Pro_*AtSWEET4*_-*GUS* analysis showed *AtSWEET4* is not expressed in pollens although it is expressed in anther filaments (Fig. 5). These results indicate AtSWEET4 and AtSWEET5 function differently. AtSWEET17 is recently identified as a fructose-specific transporter mediating fructose transport across the tonoplast of *Arabidopsis* roots and leaves. Pro_*AtSWEET17*_-*GUS* analysis showed *AtSWEET17* is expressed in the cortex of roots and root tips^19^, by contrast, *AtSWEET4* is expressed in the stele of roots (Fig. 5B,C). The expression patterns of *AtSWEET17* and *AtSWEET4* in roots suggest that these two proteins function differently but coherently in position.

Many plant pathogens acquire sugars from their hosts, thus sugar efflux/influx systems may be hijacked by pathogens for their propagation. For example, *Pst* DC3000 infection highly induced mRNA levels of a set of *AtSWEET* genes including *AtSWEET4*^15^. Presumably, the induction of SWEET gene expression is to induce sugar efflux to feed bacteria in apoplasm^15^. The expression level of *VvSWEET4* was dramatically up-regulated by the infection of *Botrytis cinerea*^21^. In addition, the knockout mutants *atsweet4-1* and *-2* were found to be less susceptible to *B. cinerea*, but as susceptible as Col-0 to virulent *Pst* DC3000^21^. RNAi4-8, OE4-4, and Col-0 plants did not show difference in bacterial growth for *Pst* DC3000 (Supplementary Fig. 4D), but they were differentiated in susceptibility to nonhost bacterium *Psph* NPS3121 in that OE4-4 line was more susceptible than Col-0 (Fig. 4D). OE4-4 line accumulates more glucose and fructose which may be used as nutrients by nonhost bacteria.

In conclusion, the expression level of *AtSWEET4* in *Arabidopsis* acts as a crucial factor affecting plant phenotype. AtSWEET4 is able to transport sugars such as glucose and fructose, which is important for sugar import in axial tissues and affects the response to biotic and abiotic stresses.

## Materials and Methods

### Plant materials and growth conditions

All *Arabidopsis* plants used in this study are in Columbia-0 ecotype background. The *atsweet4-3* mutant is a homozygous T-DNA insertion mutant (SALK_200835) from Arabidopsis Biological Resource Center (ABRC). Plants were grown in small pots filled with a mix of Professional Growing Mix soil and vermiculite (3:1 ratio) or solid agar media in a controlled growth room at 22 °C day and night, and light was set at 125 μmol m^−2^ s^−1^ illumination under a 10/14-h day/night regime. For germination experiments and different treatment assays, *Arabidopsis* seeds were soaked with tap water for 2 h, excess water was removed and the wet seeds were kept in the dark at 4 °C for 4 days, then the seeds were sterilized in 5% sodium hypochloride for 8 min, and continuously washed in sterilized distilled water for 8 times. The surface-sterilized seeds were sown on half-strength Murashige and Skoog (MS) solid media (1/2 MS salts) supplemented without or with indicated concentration of sugars. Photos of seedlings were taken at the indicated time and germination rates were determined in duplicate with 30 seeds for each line per assay.

### Generation of *AtSWEET4* RNA-interference and overexpression plants

An *AtSWEET4* RNA-interference construct was used to generate RNAi transgenic plants. A 415-bp fragment of *AtSWEET4* was PCR-amplified from cDNA using high fidelity KOD polymerase (TOYOBO, Japan) with gene specific primers (Supplementary Table S1). The fragment sequence was BLASTed against NCBI database for gene specificity, and the BLAST result showed that it does not match other genes but the *AtSWEET4*. A portion of the 415-bp PCR fragment was first digested with *Nco*I and *Asc*I and inserted into the same digested pFGC5941 vector. After confirmation of the ligation, the resulting construct was cut with *Xba*I and *Bam*HI, and ligated to the other portion of the 415-bp PCR fragment cut with the same enzymes. The complete RNAi construct harboring two copies of the 415-bp fragment with opposite direction, driven by 35S promoter, was inserted into pFGC5941 vector (Selection marker: Kan^+^ resistance in bacterial, BAR^+^ resistance in plants), and transferred into the *Agrobacterium tumefaciens* strain GV3101.

To overexpress *AtSWEET4*, the full length of *AtSWEET4* CDS was amplified using high fidelity KOD polymerase (TOYOBO, Japan) with gene specific primers (Supplementary Table S1) and cut with *Xba*I and *Sac*I, and then inserted into the same cut plasmid of pBI121 (Selection marker: Kan^+^ in both bacteria and plants), from which *AtSWEET4* is expressed under the control of the 35S promoter. The resulting overexpression construct was transferred into the *Agrobacterium tumefaciens* strain GV3101.

Transformation of *Arabidopsis* wild-type Col-0 plants was performed by the floral dip method^31^, using the *Agrobacterium tumefaciens* strain GV3101 harboring the RNAi construct or overexpression construct. RNAi construct transformation resulted in 17 independent RNAi transgenic lines, of which RNAi4-8 was used for analysis, while 14 overexpression lines were obtained from overexpression construct transformation, and line OE4-4 was selected for analysis.

### Generation of Pro_*AtSWEET4*_ *-GUS* plants and localization of AtSWEET4

For Pro_*AtSWEET4*_*-GUS* transcriptional construct, *AtSWEET4* promoter fragment upstream the start codon was amplified by PCR from *Arabidopsis* genomic DNA, the resulting 1500 bp fragment was digested with *Pst*I and *Nco*I and cloned into the same sites of pCAMBIA1391Z vector. The final binary vector containing the *AtSWEET4* promoter fragment and *GUS* gene was transformed into wild-type Col-0 plants using *A. tumefaciens* strain GV3101 and floral dip method^31^. Transgenic plants were identified on 1/2 MS media containing 50 μg mL^−1^ kanamycin. Expression of the *GUS* gene in the transgenic plants was analyzed by histochemical staining following the standard procedures with minor changes^32^. Transgenic plant seedlings or detached plant tissues were prefixed in ice-cold 90% (v/v) acetone for 20 min and washed three times with 100 mM phosphate buffer (pH 7.2) for 5 min each. Staining strength was controlled by modulating incubation time at 37 °C. After staining, tissues were cleared by placing in 70% and 90% (v/v) ethanol for several times as necessary.

For constructing the 35S promoter controlled *AtSWEET4-YFP* construct, the full length of *AtSWEET4* cDNA was amplified by high fidelity KOD polymerase, the PCR fragment was cut with *Xho*I and *ECo*RI and inserted into the same sites of pSAT6*-YFP* vector. *Arabidopsis* protoplasts were isolated following the methods as described^33^. The pSAT6*-AtSWEET4-YFP* construct was transiently expressed in the protoplasts by polyethylene glycerol (PEG)-mediated transformation as described^34^. YFP fluorescence was detected using a ZEISS-510 Meta confocal microscope, the images were coded yellow for YFP and red for chlorophyll autofluorescence. Sequences of primers were listed in Supplementary Table S1.

### Determination of relative electrolyte leakage

The relative electrical conductivities of wild type and transgenic *Arabidopsis* plants were assessed using the protocol as described^35^. Briefly, 100 mg of leaves were placed in 25 mL distilled water and shaken on a gyratory shaker at 200 rpm for 2 h at room temperature; the same amount of distilled water was used as blank control. The initial conductivities of samples and blank control were recorded as E1 and EB1, respectively. After recording, all samples were boiled for 10 min to induce electrolyte leakage, and cooled down at room temperature. The electrolyte conductivities of samples and blank control were recorded as E2 and EB2, respectively. The relative electrical conductivity was calculated as the ratio of (E1-EB1)/(E2-EB2). All experiments were carried out in triplicate.

### Glucose and fructose quantification

Glucose and fructose were extracted from *Arabidopsis* plant leaves and quantified using a new approach as described^36^. Fifty milligrams of fully expanded rosette leaves from 3-week-old *Arabidopsis* plants were harvested at the end of night. The samples were put in a tube and 500 μL Me_2_SO was added to extract the saccharides with Fastprep shaking 10 times (10 s each time) at 4 °C. The homogenate was agitated for 1 h at 4 °C and then centrifuged at 10000 rpm for 5 min. The supernatant was transferred to a 2 mL tube containing 30 μL 1-methylimidazole and 150 μL acetic anhydride. After stirring for 10 min, 600 μL ddH_2_O was added to the tube, and then the mixture was extracted with 200 μL CH_2_Cl_2_. One microliter of the reaction mixture was injected into the gas chromatograph-mass spectrometry (GC-MS) for analysis, and glucose and fructose standards were used as internal references.

### Chlorophyll quantification

The chlorophyll content was determined according to the described method with some modifications^37^. Briefly, 500 mg of fully expanded rosette leaves were harvested at the daytime and excised into strips and extracted with 25 mL extraction buffer (ethanol: acetone = 1: 1) on the shaker. The absorption values of the supernatants were measured with spectrophotometer at 663 nm and 645 nm wave length and the contents of chlorophyll a and b were calculated.

### Quantitative RT-PCR analysis

For quantitative RT-PCR, total RNA was isolated from different tissues of the wild type and transgenic *Arabidopsis* plants using RNeasy Plus Kit (QIAGEN), and the iScript cDNA Synthesis Kit (Bio-Rad) was used for the synthesis of corresponding cDNAs. All procedures just followed the manufacture’s instructions. The resulting cDNAs were diluted and used as templates for quantitative RT-PCR and RT-PCR. Amplification of an *Actin* cDNA (*Actin2*, At3G18780) was used to normalize results from different samples. Quantitative PCR was performed using HotStart-IT SYBR Green qPCR Master Mix (USB) according to the manufacturer’s instructions on a 7300 PCR system (Applied Biosystems). The relative expression level was determined by comparing with the expression of *Actin2* (2^−ΔΔCt^), where Ct represents the threshold cycle^38^. The gene-specific primers were listed in Supplementary Table S1.

### Bacterial growth assay

Infection of *Arabidopsis* with *P. syringae* was performed on 5-week-old soil-grown plants. *P. syringae* strains were grown at 28 °C in King’s medium B as described^39^ with appropriate antibiotics. Cultures were washed with 10 mM MgCl_2_ and leaves were infiltrated on the abaxial surface with a needleless syringe. Growth of *P. syringae* bacteria in leaves was determined as described^40^.

### Yeast EBY.VW4000 strain growth complementation assay

AtSWEET4 coding sequence was cloned into vector pDR196, the resulting construct was introduced into yeast hexose transport mutant EBY.VW4000 strain^41^. The yeast transformants were first grown on selective synthetic complete medium (without uracil) with 2% maltose as the sole carbon source. Drop tests were used to assess the growth of transformed yeast in 2% glucose and 1% fructose solid SD media with a serial of dilutions (OD_500_ = 1, 10^−1^, 10^−2^, 10^−3^, 10^−4^). The transformants with empty vector was used as a negative control. All transformants were grown at 30 °C for 3 days.

## Additional Information

**How to cite this article**: Liu, X. *et al.* AtSWEET4, a hexose facilitator, mediates sugar transport to axial sinks and affects plant development. *Sci. Rep.* **6**, 24563; doi: 10.1038/srep24563 (2016).

## Supplementary Material

Supplementary Information
