# Supplementary material for: AtSWEET4, a hexose facilitator, mediates sugar transport to axial sinks and affects plant development
Source: Sci Rep. 2016 Apr 22;6:24563. doi: 10.1038/srep24563 (PMC4840376; doi:10.1038/srep24563)
Supplement: Supplementary Information [file srep24563-s1.pdf]

Supplementary Information

AtSWEET4, a hexose facilitator, mediates sugar transport to axial sinks and affects plant development

Xiaozhu Liu[1], Yan Zhang[2], Chao Yang[1], ZhihongTian[2*] & Jianxiong Li[1, 2*]

[1]Key Laboratory of South China Agricultural Plant Molecular Analysis and Genetic Improvement, and Guangdong Provincial Key Laboratory of Applied Botany, South China Botanical Garden, Chinese Academy of Sciences, Guangzhou 510650, China. [2]College of Life Science, Yangtze University, Jingzhou 434025, China (Y.Z., Z.H.T., J.X.L)

*Correspondence and request for materials should be addressed to J.X.L(jxli@scbg.ac.cn) or Z.H.T(zhtian@yangtzeu.edu.cn)

Supplementary Figure Legends

Supplementary Figure 1. **Phenotypic characterization of *AtSWEET4* transgenic plants.** (A) Leaves from two RNAi lines RNAi4-8 and RNAi4-5 and two OE lines OE4-4 and OE4-2. Scale bars: 1 cm. (B) The wet biomasses of Col-0, RNAi4-8 and OE4-4. *, p value < 0.05; **, p value < 0.01.

Supplementary Figure 2. **Characteristation of *atsweet4-3* mutant.** (A) Schematic structure of *AtSWEET4* and the position of the T-DNA insertion in *atsweet4-3* (SALK_200835). Exons are shown as filled boxes, introns as lines, and 5' and 3' UTR as dashed lines. (B) Seedlings of Col-0, RNAi4-8, OE4-4, and *atsweet4-3* grown in soil. (C) Phenotypes of 4-week-old Col-0 and *atsweet4-3* plants, and young seedlings of *atsweet4-3*. (D) RT-PCR detection of *AtSWEET4* expression in Col-0, RNAi4-8, OE4-4, and *atsweet4-3* plants. (E) Phenotype of F_1 progeny from the cross of OE4-4 and RNAi4-8. (F) Phenotype of F_1 progeny from the cross of OE4-4 and *atsweet4-3*. Scale bars: 1 cm in (B) and 1 mm in (E and F).

Supplementary Figure 3. **Osmotic control for seedling growth on 1/2 MS media.** Seedlings of Col-0, RNAi4-8, OE4-4, and *atsweet4* on 1/2 MS media supplemented with 1% sorbitol (A) and 6% sorbitol (B). (C) Responses of Col-0, RNAi4-8, OE4-4 and *atsweet4-3* seedlings to 2 μM ABA. (D) Starch accumulation in Col-0, RNAi4-8, and OE4-4 plants at the end of the light and dark periods.

Supplementary Figure 4. **Characterization of *AtSWEET4*.** (A) Expression levels of *AtSWEET4* in different tissues detected by qRT-PCR. Amplification of an *Actin* cDNA (*Actin2*, At3G18780) was used to normalize the results from different samples. (B) Expression levels of *AtSWEET5* in the flowers of Col-0, RNAi4-8, and OE4-4. (C) Growth of virulent bacterium *Pst* DC3000 in the leaves of Col-0, RNAi4-8, and OE4-4.

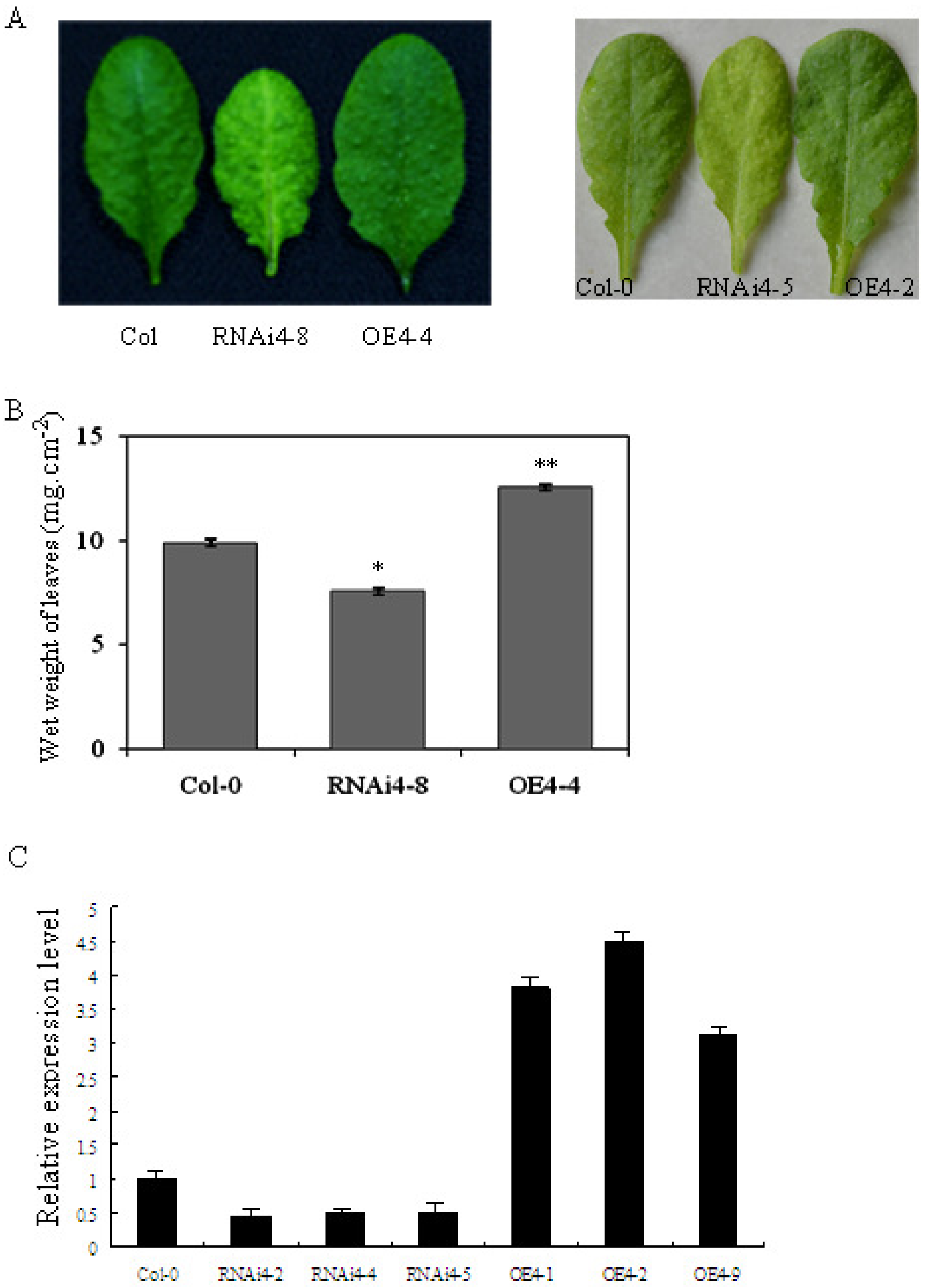

Supplementary Figure 1

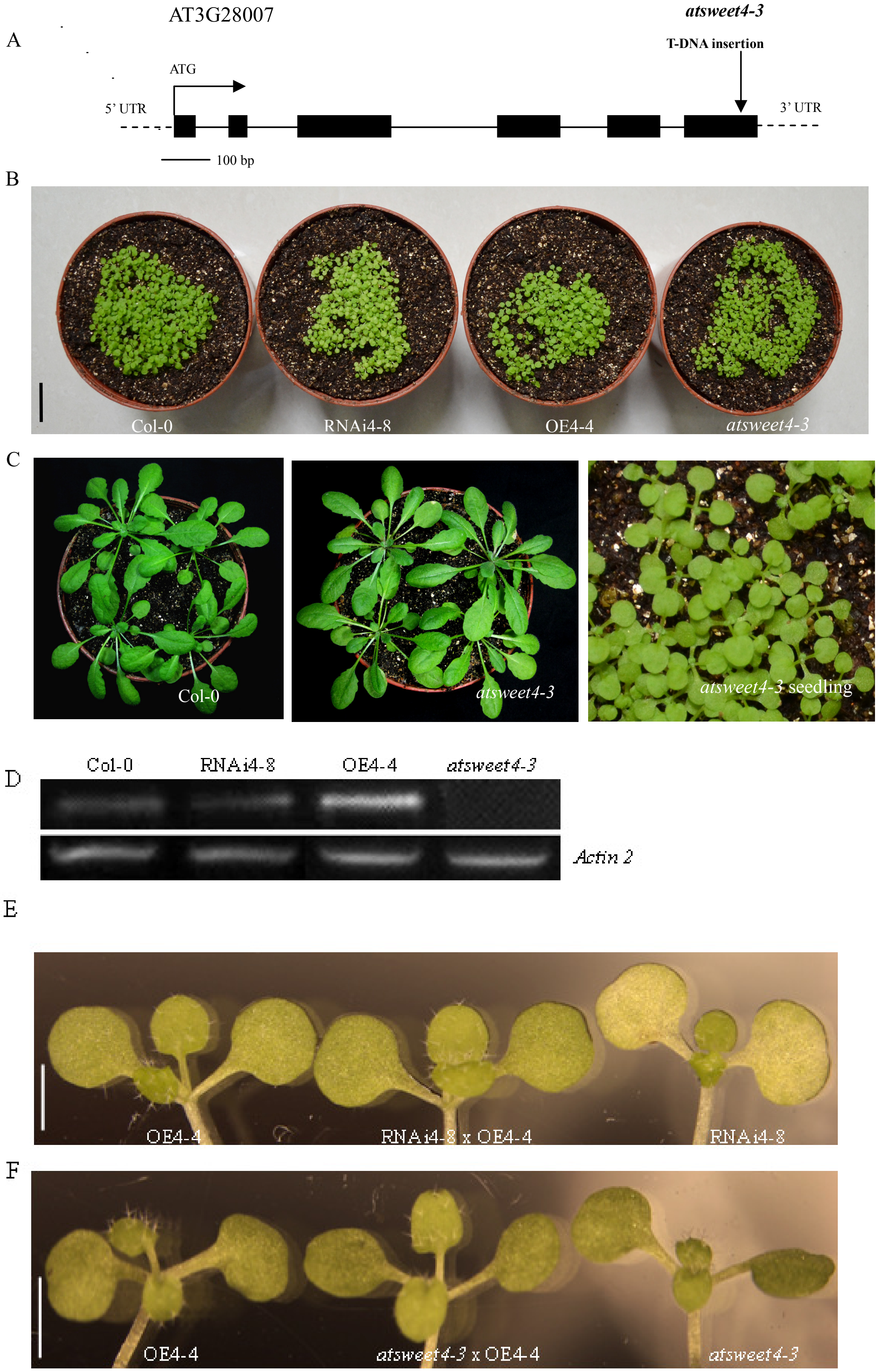

Supplementary Figure 2

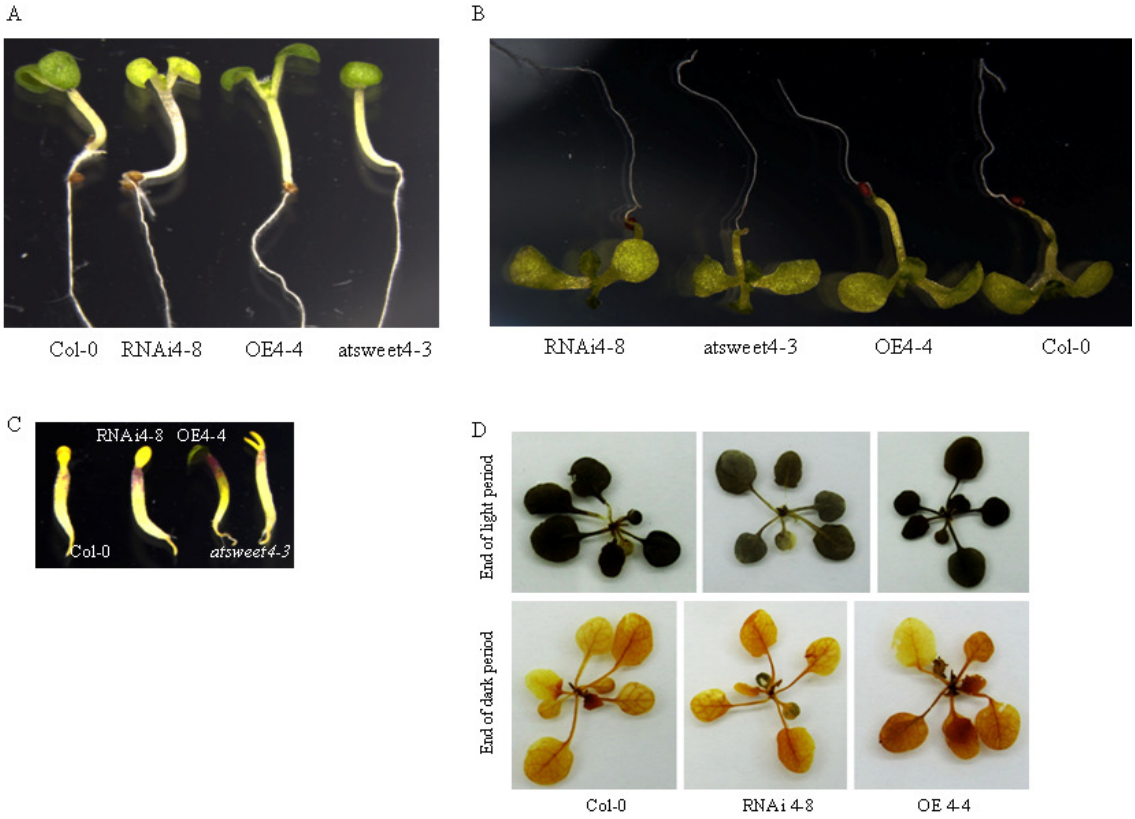

Supplementary Figure 3

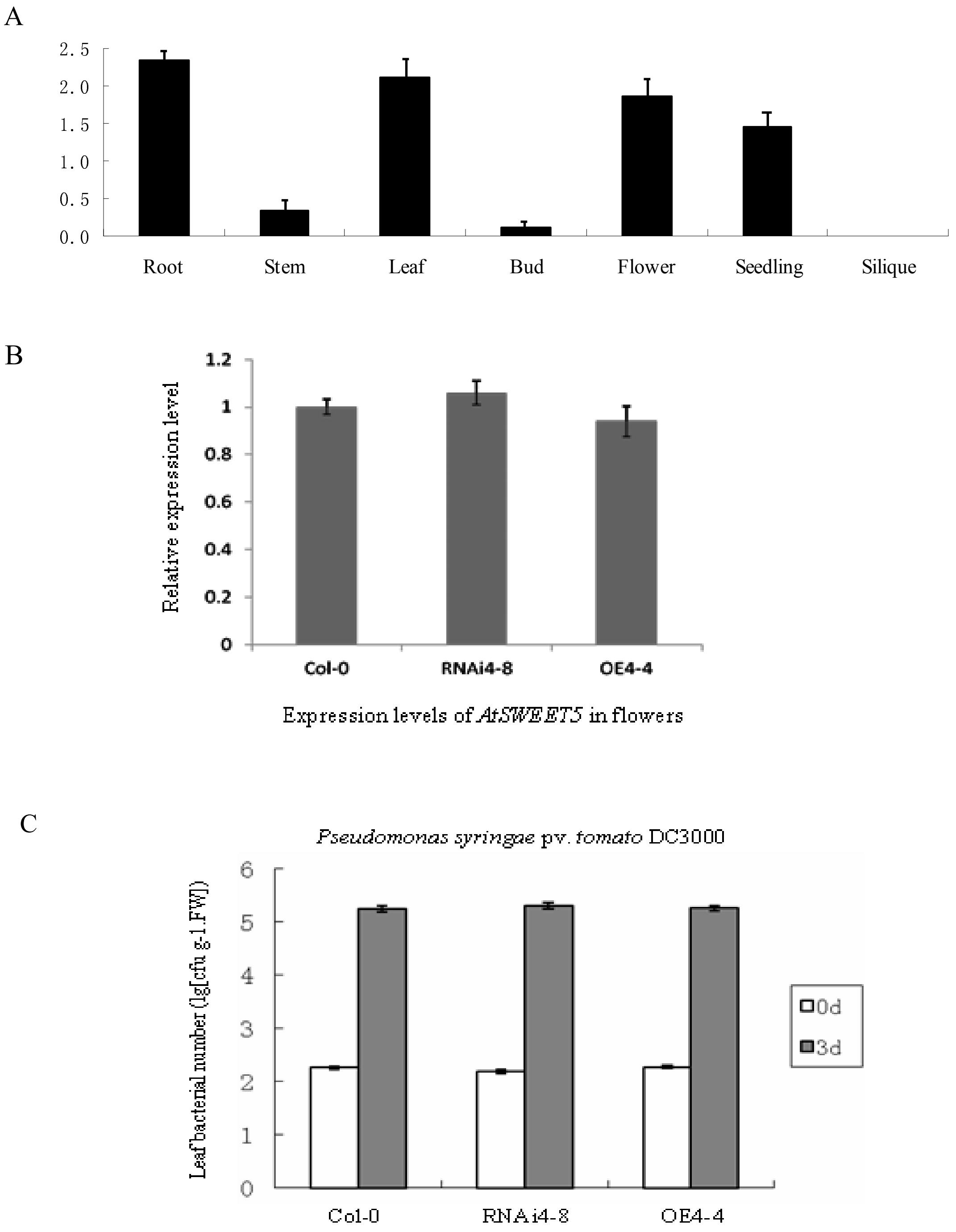

Supplementary Figure 4

Table S1. Primers used for plasmid construction and quantitative RT-PCR.

Primer name	Forward primer (5' to 3')	Reverse primer (5' to 3')
OE-SWEET4	CTCTAGAATGGTTAACGCT ACAGTTGCGAG	CGAGCTCTCAAGCTGAAAC TCGTTTAGCTTG
RNAi-SWEET4	CTCTAGACCATGGGGAGA GATGGTGTTTGTAGGAA	CGGGATCCGGCGCGCCCTTG TCCACTGTTGCCACTAAG
$P_{AtSWEET4}$-GUS	ACTGCAGCCCATAGAGTAA AAAGTGAATG	ATGCCATGGTTCACTTCAAA AGAAAAATCCG
AtSWEET4-EYFP	CCGCTCGAG CATGGTTAAC GCTACAGTTGCG	CCGGAATTC AGCTGAAACT CGTTTAGCTTG
AtSWEET4-qPCR	CCATCATGAGTAAGGTGAT CAAGA	AAAATGAAAAGGTCGAACT TAATAAGTG
AtSWEET5-qPCR	GACCCTTATATTTTGATTCC AAATGGT	GCCAAGTTCGATTCCAGCATTC
AtGSA-AT1 (At5g63570)	ATTCTTGTGTAGTGAGTTGAA	GGCAAATCGGTTTGGTATTA
AtGSA-AT2 (At3g48730)	CTCTAAAATGTCTCTGGAAG	GTCAACAAATCTTCATAGAC
Actin2-qPCR	GCACCCTGTTCTTCTTACCG	AACCCTCGTAGATTGGCACA
